# 4,4′-Diazenediyldipyridinium 4-(4-pyridyldiazen­yl)pyridinium octa­cyanidotungstate(V) dihydrate

**DOI:** 10.1107/S1600536809030153

**Published:** 2009-08-08

**Authors:** Su-Yan Qian, Wen-Yan Liu, Ai-Hua Yuan

**Affiliations:** a School of Material Science and Engineering, Jiangsu University of Science and Technology, Zhenjiang 212003, People’s Republic of China

## Abstract

The asymmetric unit of the title complex, (C_10_H_10_N_4_)(C_10_H_9_N_4_)[W(CN)_8_]·2H_2_O, contains two 4,4′-diazenediyldipyridinium, [H_2_(4,4′-azpy)]^2+^, two 4-(4-pyridyldiazen­yl)pyridinium, [H(4,4′-azpy)]^+^, cations, two [W^V^(CN)_8_]^3−^ anions, and four uncoordinated water mol­ecules. Each of the W centers is coordinated by eight CN groups in a slightly distorted square-anti­prismatic geometry. In the crystal structure, intra- and inter­molecular N—H⋯O, N—H⋯N and O—H⋯N hydrogen bonds link the cations and anions in an alternating fashion, forming a two-dimensional layered structure, in which they are further linked through the very weak π–π stacking inter­actions [shortest distance = 4.640 (2) Å] and van der Waals forces between adjacent layers, forming a three-dimensional supra­molecular network.

## Related literature

For general background to heterometallic cyanido-bridged 4*f*–4*d* or 4*f*–5*d* assemblies, see: Chelebaeva *et al.* (2008 ▶); Ikeda *et al.* (2005 ▶); Kosaka *et al.* (2007 ▶); Matoga *et al.* (2005 ▶); Przychodzeń *et al.* (2007 ▶); Wang *et al.* (2006 ▶). For a related structure, see: Liu *et al.* (2008 ▶).
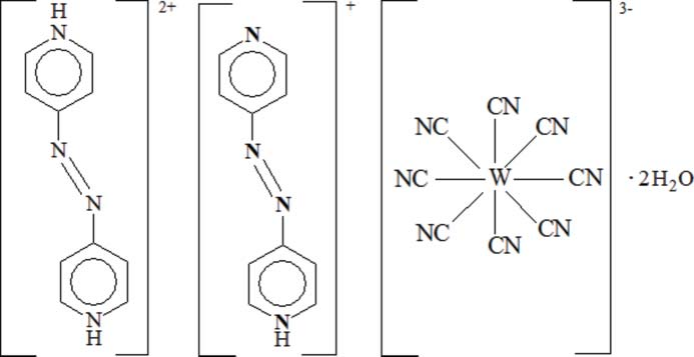


## Experimental

### 

#### Crystal data

(C_10_H_10_N_4_)(C_10_H_9_N_4_)[W(CN)_8_]·2H_2_O
*M* *_r_* = 799.46
Orthorhombic, 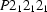

*a* = 12.7310 (16) Å
*b* = 16.499 (2) Å
*c* = 30.704 (4) Å
*V* = 6449.3 (14) Å^3^
*Z* = 8
Mo *K*α radiation
μ = 3.64 mm^−1^
*T* = 291 K
0.28 × 0.22 × 0.20 mm

#### Data collection

Bruker SMART APEX diffractometer
Absorption correction: multi-scan (*SADABS*; Bruker, 2004 ▶) *T*_min_ = 0.391, *T*_max_ = 0.483
39717 measured reflections
12501 independent reflections
8553 reflections with *I* > 2σ(*I*)
*R*_int_ = 0.048

#### Refinement

*R*[*F* ^2^ > 2σ(*F* ^2^)] = 0.047
*wR*(*F* ^2^) = 0.102
*S* = 1.03
12501 reflections
847 parameters
H-atom parameters constrained
Δρ_max_ = 1.35 e Å^−3^
Δρ_min_ = −1.71 e Å^−3^
Absolute structure: Flack (1983 ▶), 5514 Friedel pairs
Flack parameter: 0.045 (10)

### 

Data collection: *SMART* (Bruker, 2004 ▶); cell refinement: *SAINT* (Bruker, 2004 ▶); data reduction: *SAINT*; program(s) used to solve structure: *SHELXS97* (Sheldrick, 2008 ▶); program(s) used to refine structure: *SHELXL97* (Sheldrick, 2008 ▶); molecular graphics: *SHELXTL* (Sheldrick, 2008 ▶) and *DIAMOND* (Brandenburg, 2006 ▶); software used to prepare material for publication: *SHELXL97*.

## Supplementary Material

Crystal structure: contains datablocks I, global. DOI: 10.1107/S1600536809030153/hk2738sup1.cif

Structure factors: contains datablocks I. DOI: 10.1107/S1600536809030153/hk2738Isup2.hkl

Additional supplementary materials:  crystallographic information; 3D view; checkCIF report

## Figures and Tables

**Table 1:** Hydrogen-bond geometry (Å, °)

*D*—H⋯*A*	*D*—H	H⋯*A*	*D*⋯*A*	*D*—H⋯*A*
N20—H20*A*⋯O2	0.86	1.83	2.671 (10)	166
N21—H21*A*⋯N17^i^	0.86	1.76	2.586 (10)	162
N24—H24⋯O3	0.86	1.73	2.560 (10)	162
N28—H28*A*⋯O4^ii^	0.86	1.84	2.670 (10)	161
N32—H32*A*⋯O1^iii^	0.86	1.88	2.719 (10)	166
O2—H2*B*⋯N3^iv^	0.85	2.47	2.914 (10)	113
O1—H1*B*⋯N7	0.85	2.58	3.076 (10)	119
O4—H4*C*⋯N4^v^	0.85	2.25	2.845 (9)	127
O3—H3*C*⋯N14^v^	0.85	2.50	2.960 (9)	115
O4—H4*B*⋯N10^vi^	0.85	2.47	2.913 (9)	114

Symmetry codes: (i) 
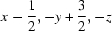
; (ii) 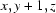; (iii) 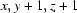; (iv) 
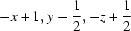
; (v) 
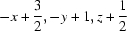
; (vi) 
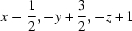
.

**Fig. 1.**
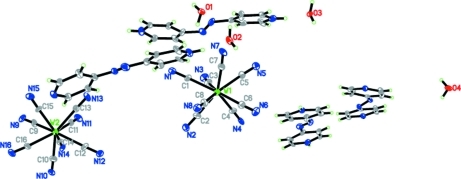
The molecular structure of the title molecule with the partial atom-numbering scheme. Displacement ellipsoids are drawn at the 30% probability level. The atoms of [H2(4,4'-azpy)]2+ and [H(4,4'-azpy)]+ cations are not labelled for clarity.

**Fig. 2.**
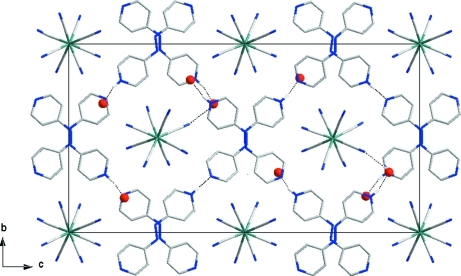
A partial packing digaram viewed down the a axis. Hydrogen bonds are shown as dashed lines.

**Fig. 3.**
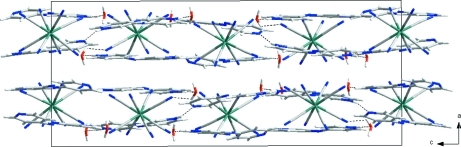
The three-dimensional supramolecular network.

## supplementary crystallographic information

### Comment

Heterometallic cyanido-bridged 4f-4d or 4f-5d assemblies are of high interest in molecular magnetism over the recent years (Chelebaeva *et al.*, 2008; Przychodzeń *et al.*, 2007; Ikeda *et al.*, 2005; Kosaka *et al.*, 2007; Matoga *et al.*, 2005; Wang *et al.*, 2006). The combination of the octacyanometalate [*M*(CN)_8_]^3-/4- ^(*M* = Mo, W, Nb) building blocks with the lanthanide ions are of interest not only in materials science but also in physics and theoretical chemistry, providing interesting cases of investigation. Herein, we employed 4,4'-azpy (4,4'-azobispyridine), [W^V^(CN)_8_]^3-^, and Ce^3+^ as building blocks in order to obtain a new lanthanide-containing octacyanotungstate(V)-based magnet. However, the unexpected supramolecular title complex without Ce^3+^ was obtained. We report herein its crystal structure.

The asymmetric unit of the title complex contains two 4,4'-diazenediyldi- pyridinium, [H_2_(4,4'-azpy)]^2+^, and two (4-pyridyldiazenyl)pyridinium, [H(4,4'-azpy)]^+^, cations, two [W^V^(CN)_8_]^3-^ anions, and four uncoordinated water molecules (Fig. 1). Each of the W centers is coordinated by eight CN groups in a slightly distorted square antiprism. The average W-C bond distances are 2.153 (8) Å (for W1) and 2.178 (8) Å (for W2).

In the crystal structure, intra- and intermolecular N-H···O, N-H···N and O-H···N hydrogen bonds (Table 1) link the [H_2_(4,4'-azpy)]^2+^ and [H(4,4'-azpy)]^+^ cations and [W(CN)_8_]^3-^ anions in an alternating fashion to form a two-dimensional layered structure (Fig. 2), in which they are further linked through the highly weak π–π stacking interactions [shortest distance = 4.640 (2) Å] and van der Waals forces between adjacent layers to form a three-dimensional supramolecular network (Fig. 3) as in the similiar complex (C_10_H_10_N_4_)(C_10_H_9_N_4_)[Mo(CN)_8_].4H_2_O (Liu *et al.*, 2008).

### Experimental

Single crystals of the title complex were prepared at room temperature in the dark by slow diffusion of an acetonitrile solution (2 ml) containing both Ce(NO_3_)_3_.6H_2_O (21.71 mg, 0.05 mmol) and 4,4'-azpy (9.21 mg, 0.05 mmol) in an acetonitrile solution (20 ml) of [HN(n-C_4_H_9_)_3_]_3_[W(CN)_8_].4H_2_O (46.60 mg, 0.05 mmol). After two weeks, pale yellow crystals were obtained.

### Refinement

H atoms were positioned geometrically with O-H = 0.85 Å (for H_2_O), N-H = 0.86 Å (for NH) and C-H = 0.93 Å, for aromatic H atoms, respectively, and constrained to ride on their parent atoms, with U_iso_(H) = xU_eq_(C,N,O), where x = 1.5 for H_2_O H and x = 1.2 for all other H atoms.

### Crystal data

**Table:**

(C_10_H_10_N_4_)(C_10_H_9_N_4_)[W(CN)_8_]·2H_2_O	*F*(000) = 3144
*M**_r_* = 799.46	*D*_x_ = 1.647 Mg m^−^^3^
Orthorhombic, *P*2_1_2_1_2_1_	Mo *K*α radiation, λ = 0.71073 Å
Hall symbol: P 2ac 2ab	Cell parameters from 3042 reflections
*a* = 12.7310 (16) Å	θ = 2.4–23.3°
*b* = 16.499 (2) Å	µ = 3.64 mm^−^^1^
*c* = 30.704 (4) Å	*T* = 291 K
*V* = 6449.3 (14) Å^3^	Pale, yellow
*Z* = 8	0.28 × 0.22 × 0.20 mm

### Data collection

**Table:**

Bruker SMART APEX diffractometer	12501 independent reflections
Radiation source: fine-focus sealed tube	8553 reflections with *I* > 2σ(*I*)
graphite	*R*_int_ = 0.048
φ and ω scans	θ_max_ = 26.0°, θ_min_ = 1.3°
Absorption correction: multi-scan (*SADABS*; Bruker, 2004)	*h* = −15→15
*T*_min_ = 0.391, *T*_max_ = 0.483	*k* = −20→16
39717 measured reflections	*l* = −25→37

### Refinement

**Table:**

Refinement on *F*^2^	Secondary atom site location: difference Fourier map
Least-squares matrix: full	Hydrogen site location: inferred from neighbouring sites
*R*[*F*^2^ > 2σ(*F*^2^)] = 0.047	H-atom parameters constrained
*wR*(*F*^2^) = 0.102	*w* = 1/[σ^2^(*F*_o_^2^) + (0.05*P*)^2^] where *P* = (*F*_o_^2^ + 2*F*_c_^2^)/3
*S* = 1.03	(Δ/σ)_max_ < 0.001
12501 reflections	Δρ_max_ = 1.35 e Å^−^^3^
847 parameters	Δρ_min_ = −1.71 e Å^−^^3^
0 restraints	Absolute structure: Flack (1983), 5514 Friedel pairs
Primary atom site location: structure-invariant direct methods	Flack parameter: 0.045 (10)

### Special details

**Table:**

Geometry. All e.s.d.'s (except the e.s.d. in the dihedral angle between two l.s. planes) are estimated using the full covariance matrix. The cell e.s.d.'s are taken into account individually in the estimation of e.s.d.'s in distances, angles and torsion angles; correlations between e.s.d.'s in cell parameters are only used when they are defined by crystal symmetry. An approximate (isotropic) treatment of cell e.s.d.'s is used for estimating e.s.d.'s involving l.s. planes.
Refinement. Refinement of *F*^2^ against ALL reflections. The weighted *R*-factor *wR* and goodness of fit *S* are based on *F*^2^, conventional *R*-factors *R* are based on *F*, with *F* set to zero for negative *F*^2^. The threshold expression of *F*^2^ > σ(*F*^2^) is used only for calculating *R*-factors(gt) *etc*. and is not relevant to the choice of reflections for refinement. *R*-factors based on *F*^2^ are statistically about twice as large as those based on *F*, and *R*- factors based on ALL data will be even larger.

### Fractional atomic coordinates and isotropic or equivalent isotropic displacement parameters (Å^2^)

**Table:**

	*x*	*y*	*z*	*U*_iso_*/*U*_eq_	
C1	0.6378 (6)	0.5473 (4)	0.1970 (3)	0.0297 (18)	
C2	0.8138 (6)	0.6063 (5)	0.2335 (3)	0.0313 (18)	
C3	0.6200 (5)	0.5979 (5)	0.2747 (3)	0.0336 (19)	
C4	0.7952 (5)	0.5479 (5)	0.3113 (3)	0.0342 (19)	
C5	0.6367 (6)	0.4511 (5)	0.3072 (3)	0.038 (2)	
C6	0.8309 (6)	0.4089 (5)	0.2733 (3)	0.0362 (19)	
C7	0.6359 (7)	0.4012 (5)	0.2289 (3)	0.038 (2)	
C8	0.8104 (6)	0.4661 (5)	0.1968 (3)	0.0324 (18)	
C9	0.6935 (6)	1.1035 (5)	−0.0145 (3)	0.037 (2)	
C10	0.8908 (6)	1.0955 (5)	0.0232 (3)	0.040 (2)	
C11	0.6993 (6)	1.0445 (4)	0.0620 (3)	0.0301 (17)	
C12	0.8724 (6)	0.9562 (5)	0.0597 (3)	0.036 (2)	
C13	0.6913 (6)	0.8979 (5)	0.0264 (3)	0.0353 (19)	
C14	0.8822 (6)	0.9009 (5)	−0.0216 (3)	0.0318 (18)	
C15	0.6849 (6)	0.9547 (5)	−0.0491 (3)	0.0329 (19)	
C16	0.8540 (6)	1.0422 (5)	−0.0566 (3)	0.035 (2)	
C17	0.7019 (6)	0.6236 (4)	−0.1048 (3)	0.0340 (18)	
H17	0.7053	0.6073	−0.1337	0.041*	
C18	0.6731 (6)	0.5699 (5)	−0.0733 (3)	0.0355 (19)	
H18	0.6568	0.5168	−0.0808	0.043*	
C19	0.6678 (6)	0.5951 (5)	−0.0291 (3)	0.0339 (18)	
C20	0.6898 (6)	0.6746 (5)	−0.0179 (3)	0.0360 (18)	
H20	0.6852	0.6916	0.0109	0.043*	
C21	0.7189 (6)	0.7285 (5)	−0.0504 (3)	0.0344 (18)	
H21	0.7334	0.7821	−0.0433	0.041*	
C22	0.6516 (7)	0.2790 (5)	0.0626 (3)	0.043 (2)	
H22	0.6549	0.2236	0.0574	0.052*	
C23	0.6590 (6)	0.3321 (5)	0.0289 (3)	0.038 (2)	
H23	0.6672	0.3135	0.0005	0.045*	
C24	0.6540 (6)	0.4142 (5)	0.0375 (3)	0.038 (2)	
C25	0.6430 (7)	0.4397 (6)	0.0816 (3)	0.047 (2)	
H25	0.6426	0.4948	0.0880	0.056*	
C26	0.6337 (6)	0.3887 (5)	0.1125 (3)	0.040 (2)	
H26	0.6229	0.4067	0.1409	0.048*	
C27	0.3214 (7)	0.6179 (5)	0.1410 (3)	0.043 (2)	
H27	0.3126	0.6004	0.1124	0.051*	
C28	0.3426 (6)	0.5650 (5)	0.1707 (3)	0.039 (2)	
H28	0.3508	0.5109	0.1628	0.047*	
C29	0.3538 (6)	0.5877 (5)	0.2162 (3)	0.037 (2)	
C30	0.3463 (6)	0.6675 (5)	0.2279 (3)	0.0355 (19)	
H30	0.3544	0.6835	0.2568	0.043*	
C31	0.3253 (6)	0.7263 (6)	0.1939 (3)	0.039 (2)	
H31	0.3210	0.7811	0.2009	0.047*	
C32	0.3825 (6)	0.2770 (5)	0.3084 (3)	0.037 (2)	
H32	0.3948	0.2224	0.3028	0.045*	
C33	0.3711 (7)	0.3313 (5)	0.2739 (3)	0.046 (2)	
H33	0.3769	0.3135	0.2453	0.055*	
C34	0.3510 (6)	0.4119 (5)	0.2828 (3)	0.035 (2)	
C35	0.3408 (6)	0.4365 (5)	0.3262 (3)	0.040 (2)	
H35	0.3246	0.4902	0.3326	0.047*	
C36	0.3540 (7)	0.3845 (5)	0.3579 (4)	0.045 (2)	
H36	0.3486	0.4027	0.3865	0.054*	
C37	0.6111 (6)	0.7734 (5)	0.6976 (3)	0.0336 (19)	
H37	0.6148	0.7179	0.7029	0.040*	
C38	0.6055 (6)	0.8260 (5)	0.7325 (3)	0.038 (2)	
H38	0.6045	0.8064	0.7608	0.046*	
C39	0.6013 (6)	0.9083 (5)	0.7244 (3)	0.037 (2)	
C40	0.6034 (6)	0.9347 (6)	0.6811 (3)	0.039 (2)	
H40	0.6016	0.9901	0.6757	0.047*	
C41	0.6078 (6)	0.8834 (5)	0.6467 (3)	0.0330 (18)	
H41	0.6086	0.9030	0.6183	0.040*	
C42	0.6139 (6)	1.1089 (5)	0.8685 (3)	0.037 (2)	
H42	0.6108	1.0914	0.8973	0.045*	
C43	0.6087 (6)	1.0550 (5)	0.8382 (3)	0.038 (2)	
H43	0.6027	1.0002	0.8451	0.046*	
C44	0.6124 (6)	1.0821 (5)	0.7922 (3)	0.035 (2)	
C45	0.6253 (6)	1.1616 (5)	0.7834 (3)	0.036 (2)	
H45	0.6312	1.1788	0.7547	0.043*	
C46	0.6301 (6)	1.2177 (5)	0.8166 (3)	0.036 (2)	
H46	0.6372	1.2727	0.8107	0.043*	
C47	0.4424 (8)	0.7636 (6)	0.9344 (3)	0.048 (2)	
H47	0.4595	0.7095	0.9390	0.058*	
C48	0.4385 (6)	0.8192 (5)	0.9690 (3)	0.043 (2)	
H48	0.4536	0.8027	0.9973	0.052*	
C49	0.4116 (5)	0.8998 (5)	0.9601 (3)	0.035 (2)	
C50	0.3877 (6)	0.9236 (6)	0.9184 (3)	0.043 (2)	
H50	0.3682	0.9771	0.9134	0.052*	
C51	0.3917 (5)	0.8720 (5)	0.8846 (3)	0.036 (2)	
H51	0.3762	0.8891	0.8565	0.043*	
C52	0.3989 (7)	1.2149 (6)	1.0509 (3)	0.040 (2)	
H52	0.3970	1.2702	1.0451	0.048*	
C53	0.4044 (5)	1.1586 (4)	1.0177 (3)	0.0294 (18)	
H53	0.4041	1.1770	0.9891	0.035*	
C54	0.4104 (6)	1.0752 (5)	1.0252 (3)	0.038 (2)	
C55	0.4061 (7)	1.0507 (5)	1.0724 (3)	0.042 (2)	
H55	0.4091	0.9962	1.0799	0.050*	
C56	0.3982 (6)	1.1049 (5)	1.1021 (3)	0.040 (2)	
H56	0.3936	1.0879	1.1309	0.047*	
N1	0.5960 (5)	0.5701 (4)	0.1664 (2)	0.0311 (15)	
N2	0.8569 (6)	0.6651 (4)	0.2237 (3)	0.0438 (18)	
N3	0.5657 (5)	0.6483 (5)	0.2883 (3)	0.0446 (19)	
N4	0.8368 (5)	0.5730 (4)	0.3427 (2)	0.0308 (15)	
N5	0.5944 (5)	0.4196 (4)	0.3371 (2)	0.0376 (17)	
N6	0.8863 (5)	0.3586 (4)	0.2865 (2)	0.0353 (16)	
N7	0.5832 (5)	0.3496 (4)	0.2153 (2)	0.0365 (17)	
N8	0.8538 (5)	0.4451 (4)	0.1666 (3)	0.0368 (17)	
N9	0.6442 (5)	1.1600 (4)	−0.0255 (2)	0.0358 (16)	
N10	0.9452 (5)	1.1469 (4)	0.0326 (3)	0.0400 (18)	
N11	0.6588 (5)	1.0669 (4)	0.0903 (3)	0.0444 (19)	
N12	0.9160 (5)	0.9315 (4)	0.0883 (2)	0.0348 (16)	
N13	0.6466 (6)	0.8433 (4)	0.0388 (2)	0.0411 (18)	
N14	0.9354 (5)	0.8508 (4)	−0.0345 (2)	0.0328 (16)	
N15	0.6319 (5)	0.9307 (4)	−0.0754 (3)	0.0392 (18)	
N16	0.8838 (6)	1.0715 (4)	−0.0877 (3)	0.046 (2)	
N17	0.7265 (5)	0.7036 (4)	−0.0933 (2)	0.0335 (15)	
N18	0.6520 (6)	0.5431 (5)	0.0041 (3)	0.0310 (19)	
N19	0.6672 (7)	0.4666 (5)	0.0023 (4)	0.046 (2)	
N20	0.6395 (6)	0.3066 (4)	0.1039 (3)	0.039 (2)	
H20A	0.6355	0.2726	0.1250	0.047*	
N21	0.3112 (6)	0.7012 (4)	0.1502 (3)	0.0408 (17)	
H21A	0.2969	0.7355	0.1300	0.049*	
N22	0.3621 (6)	0.5397 (5)	0.2529 (3)	0.036 (2)	
N23	0.3483 (7)	0.4622 (5)	0.2472 (4)	0.045 (2)	
N24	0.3757 (6)	0.3036 (4)	0.3505 (3)	0.042 (2)	
H24	0.3847	0.2707	0.3719	0.051*	
N25	0.6113 (5)	0.8007 (4)	0.6551 (3)	0.0384 (18)	
H25A	0.6135	0.7666	0.6339	0.046*	
N26	0.6018 (6)	0.9564 (5)	0.7602 (3)	0.032 (2)	
N27	0.5977 (7)	1.0325 (5)	0.7605 (3)	0.042 (3)	
N28	0.6237 (5)	1.1889 (4)	0.8611 (3)	0.0406 (18)	
H28A	0.6261	1.2224	0.8825	0.049*	
N29	0.4199 (5)	0.7923 (4)	0.8931 (2)	0.0364 (17)	
N30	0.4065 (5)	0.9466 (4)	0.9941 (3)	0.0328 (18)	
N31	0.4201 (5)	1.0249 (4)	0.9923 (3)	0.0333 (19)	
N32	0.3964 (5)	1.1847 (4)	1.0941 (2)	0.0324 (16)	
H32A	0.3935	1.2182	1.1155	0.039*	
H2B	0.5658	0.1548	0.1525	0.049*	
H2C	0.6739	0.1518	0.1581	0.049*	
O1	0.4023 (4)	0.3080 (3)	0.15273 (19)	0.0362 (14)	
H1B	0.4589	0.3347	0.1495	0.054*	
H1C	0.3500	0.3393	0.1489	0.054*	
O2	0.6195 (5)	0.1824 (4)	0.1591 (2)	0.0351 (16)	
O3	0.3947 (4)	0.1827 (3)	0.4021 (2)	0.0330 (15)	
H3B	0.3470	0.1481	0.3969	0.050*	
H3C	0.4546	0.1610	0.3984	0.050*	
O4	0.6277 (4)	0.3185 (4)	0.9132 (2)	0.0415 (15)	
H4B	0.5687	0.3385	0.9059	0.062*	
H4C	0.6767	0.3445	0.9007	0.062*	
W1	0.721961 (19)	0.50218 (3)	0.253569 (10)	0.03326 (9)	
W2	0.783580 (19)	1.00055 (3)	0.003054 (9)	0.03288 (8)	

### Atomic displacement parameters (Å^2^)

**Table:**

	*U*^11^	*U*^22^	*U*^33^	*U*^12^	*U*^13^	*U*^23^
C1	0.035 (4)	0.024 (4)	0.030 (5)	0.000 (3)	−0.011 (4)	−0.009 (4)
C2	0.029 (3)	0.034 (4)	0.031 (5)	−0.006 (3)	0.008 (3)	−0.013 (4)
C3	0.025 (4)	0.039 (5)	0.036 (5)	0.010 (3)	0.003 (3)	−0.011 (4)
C4	0.025 (4)	0.053 (5)	0.024 (5)	−0.006 (3)	0.001 (3)	−0.012 (4)
C5	0.038 (4)	0.049 (5)	0.026 (5)	−0.001 (4)	0.005 (4)	0.000 (4)
C6	0.038 (4)	0.041 (5)	0.030 (5)	0.003 (4)	0.004 (4)	−0.004 (4)
C7	0.042 (4)	0.047 (5)	0.025 (5)	−0.011 (4)	−0.001 (4)	0.001 (4)
C8	0.043 (4)	0.023 (4)	0.032 (5)	0.002 (3)	0.005 (4)	−0.005 (3)
C9	0.040 (4)	0.033 (4)	0.038 (6)	0.010 (3)	0.010 (4)	0.014 (4)
C10	0.035 (4)	0.045 (5)	0.039 (6)	−0.010 (4)	0.003 (4)	−0.004 (4)
C11	0.036 (4)	0.025 (4)	0.029 (5)	0.001 (3)	−0.005 (4)	0.005 (3)
C12	0.033 (4)	0.039 (5)	0.037 (5)	0.007 (3)	−0.015 (4)	−0.008 (4)
C13	0.040 (4)	0.038 (5)	0.028 (5)	−0.004 (3)	−0.001 (3)	0.003 (4)
C14	0.028 (4)	0.034 (4)	0.033 (5)	−0.002 (3)	−0.001 (3)	0.000 (4)
C15	0.026 (3)	0.047 (5)	0.026 (5)	−0.001 (3)	−0.012 (3)	−0.001 (4)
C16	0.029 (4)	0.030 (4)	0.045 (6)	−0.004 (3)	0.002 (4)	0.008 (4)
C17	0.035 (4)	0.032 (4)	0.035 (5)	0.010 (3)	−0.001 (3)	0.001 (4)
C18	0.048 (5)	0.030 (4)	0.029 (5)	0.007 (3)	0.018 (4)	−0.010 (4)
C19	0.034 (4)	0.034 (4)	0.033 (5)	−0.003 (3)	0.005 (3)	−0.005 (4)
C20	0.042 (4)	0.037 (4)	0.029 (5)	−0.005 (3)	−0.005 (3)	0.000 (4)
C21	0.035 (4)	0.030 (4)	0.038 (5)	0.011 (4)	0.004 (4)	0.002 (4)
C22	0.047 (5)	0.034 (5)	0.049 (6)	−0.004 (4)	0.027 (4)	0.003 (4)
C23	0.043 (4)	0.027 (4)	0.044 (6)	−0.012 (3)	−0.023 (4)	0.008 (4)
C24	0.029 (4)	0.032 (4)	0.051 (6)	0.008 (3)	−0.004 (4)	0.010 (4)
C25	0.058 (5)	0.040 (5)	0.041 (6)	−0.001 (4)	0.002 (5)	0.001 (5)
C26	0.025 (4)	0.034 (5)	0.060 (7)	−0.017 (3)	0.016 (4)	0.002 (4)
C27	0.046 (4)	0.031 (4)	0.052 (6)	−0.022 (4)	−0.023 (4)	0.018 (4)
C28	0.044 (4)	0.038 (5)	0.036 (5)	−0.006 (4)	−0.013 (4)	0.009 (4)
C29	0.039 (4)	0.040 (5)	0.032 (5)	0.007 (4)	−0.014 (4)	0.000 (4)
C30	0.037 (4)	0.041 (5)	0.029 (5)	−0.004 (3)	0.021 (4)	−0.005 (4)
C31	0.030 (4)	0.051 (5)	0.036 (5)	0.004 (4)	−0.001 (3)	0.007 (4)
C32	0.034 (4)	0.033 (5)	0.045 (6)	−0.010 (3)	−0.002 (4)	−0.001 (4)
C33	0.055 (5)	0.029 (5)	0.054 (7)	0.027 (4)	0.007 (4)	0.013 (4)
C34	0.034 (4)	0.027 (4)	0.045 (6)	−0.011 (3)	−0.018 (4)	0.001 (4)
C35	0.037 (4)	0.031 (4)	0.050 (6)	−0.014 (3)	−0.012 (4)	−0.010 (4)
C36	0.044 (5)	0.030 (5)	0.061 (7)	−0.007 (4)	0.027 (5)	0.000 (4)
C37	0.036 (4)	0.025 (4)	0.040 (6)	−0.006 (3)	−0.011 (4)	−0.002 (4)
C38	0.033 (4)	0.039 (5)	0.044 (6)	−0.005 (3)	0.000 (4)	−0.011 (4)
C39	0.042 (4)	0.038 (5)	0.032 (5)	0.015 (4)	0.012 (4)	−0.008 (4)
C40	0.038 (4)	0.051 (5)	0.029 (5)	−0.021 (4)	−0.001 (3)	−0.004 (4)
C41	0.041 (4)	0.031 (4)	0.027 (5)	−0.009 (3)	−0.003 (3)	−0.011 (4)
C42	0.038 (4)	0.034 (5)	0.039 (6)	−0.001 (3)	−0.010 (4)	0.010 (4)
C43	0.033 (4)	0.031 (5)	0.051 (6)	0.011 (3)	−0.002 (4)	0.006 (4)
C44	0.030 (4)	0.029 (4)	0.047 (6)	0.010 (3)	−0.016 (4)	−0.005 (4)
C45	0.030 (4)	0.034 (4)	0.044 (6)	−0.017 (3)	0.004 (3)	−0.009 (4)
C46	0.039 (4)	0.035 (5)	0.034 (5)	−0.021 (3)	0.015 (4)	−0.004 (4)
C47	0.068 (6)	0.048 (5)	0.028 (5)	−0.010 (5)	0.008 (4)	0.004 (4)
C48	0.042 (4)	0.040 (5)	0.048 (6)	−0.003 (4)	−0.017 (4)	0.001 (4)
C49	0.021 (3)	0.038 (5)	0.046 (6)	−0.014 (3)	−0.002 (3)	0.004 (4)
C50	0.042 (4)	0.048 (5)	0.038 (6)	0.017 (4)	−0.027 (4)	−0.003 (4)
C51	0.019 (3)	0.048 (5)	0.040 (6)	0.015 (3)	0.003 (3)	−0.002 (4)
C52	0.048 (5)	0.050 (5)	0.023 (5)	−0.014 (4)	0.001 (4)	−0.004 (4)
C53	0.031 (4)	0.025 (4)	0.033 (5)	−0.001 (3)	0.005 (3)	−0.002 (3)
C54	0.026 (4)	0.043 (5)	0.044 (6)	−0.020 (3)	−0.006 (4)	0.008 (4)
C55	0.052 (5)	0.028 (5)	0.045 (6)	−0.004 (4)	0.001 (4)	0.006 (4)
C56	0.041 (4)	0.040 (5)	0.038 (6)	−0.013 (4)	0.017 (4)	0.005 (4)
N1	0.035 (3)	0.026 (3)	0.032 (4)	−0.003 (3)	0.003 (3)	−0.009 (3)
N2	0.048 (4)	0.044 (4)	0.040 (5)	−0.009 (3)	−0.002 (3)	0.008 (4)
N3	0.040 (4)	0.048 (5)	0.046 (5)	0.012 (3)	0.001 (3)	−0.009 (4)
N4	0.045 (4)	0.028 (3)	0.019 (4)	−0.013 (3)	0.011 (3)	0.000 (3)
N5	0.035 (3)	0.039 (4)	0.039 (5)	0.009 (3)	0.013 (3)	−0.002 (3)
N6	0.047 (4)	0.036 (4)	0.022 (4)	0.012 (3)	−0.006 (3)	−0.018 (3)
N7	0.038 (3)	0.028 (4)	0.043 (5)	−0.001 (3)	0.021 (3)	−0.001 (3)
N8	0.044 (4)	0.036 (4)	0.030 (4)	−0.002 (3)	−0.004 (3)	−0.011 (3)
N9	0.035 (3)	0.047 (4)	0.025 (4)	0.012 (3)	−0.010 (3)	−0.009 (3)
N10	0.037 (3)	0.041 (4)	0.042 (5)	−0.018 (3)	0.001 (3)	0.010 (3)
N11	0.040 (4)	0.037 (4)	0.057 (6)	0.006 (3)	0.021 (4)	0.003 (4)
N12	0.026 (3)	0.049 (4)	0.030 (4)	0.009 (3)	0.002 (3)	−0.003 (3)
N13	0.064 (4)	0.039 (4)	0.020 (4)	−0.015 (4)	0.017 (3)	−0.004 (3)
N14	0.028 (3)	0.033 (4)	0.038 (4)	0.010 (3)	−0.012 (3)	0.013 (3)
N15	0.035 (3)	0.039 (4)	0.044 (5)	−0.009 (3)	0.004 (3)	−0.012 (4)
N16	0.044 (4)	0.034 (4)	0.060 (6)	−0.011 (3)	0.005 (4)	0.020 (4)
N17	0.036 (3)	0.036 (4)	0.029 (4)	−0.002 (3)	0.013 (3)	−0.002 (3)
N18	0.021 (3)	0.037 (4)	0.035 (5)	0.002 (3)	0.007 (3)	0.008 (4)
N19	0.044 (5)	0.038 (4)	0.057 (7)	−0.009 (3)	−0.001 (5)	−0.001 (4)
N20	0.041 (4)	0.032 (4)	0.044 (5)	−0.002 (3)	0.007 (4)	0.007 (4)
N21	0.049 (4)	0.034 (4)	0.039 (5)	0.009 (3)	0.004 (3)	0.004 (3)
N22	0.033 (4)	0.041 (4)	0.035 (5)	−0.004 (3)	0.012 (4)	−0.001 (4)
N23	0.049 (5)	0.030 (4)	0.056 (6)	0.006 (3)	−0.028 (5)	0.008 (4)
N24	0.045 (4)	0.026 (4)	0.055 (6)	−0.010 (3)	−0.023 (4)	−0.006 (4)
N25	0.039 (4)	0.029 (4)	0.047 (5)	−0.008 (3)	−0.011 (3)	0.003 (3)
N26	0.039 (4)	0.032 (4)	0.026 (5)	0.009 (3)	−0.001 (4)	−0.005 (3)
N27	0.048 (5)	0.035 (4)	0.044 (6)	−0.016 (3)	−0.015 (4)	−0.016 (4)
N28	0.041 (4)	0.043 (4)	0.038 (5)	0.000 (3)	−0.010 (3)	−0.001 (4)
N29	0.042 (4)	0.032 (4)	0.034 (4)	−0.012 (3)	−0.013 (3)	0.003 (3)
N30	0.032 (3)	0.033 (4)	0.033 (5)	−0.013 (3)	0.003 (3)	0.002 (4)
N31	0.028 (3)	0.024 (4)	0.049 (5)	0.004 (2)	0.003 (3)	0.012 (3)
N32	0.038 (3)	0.039 (4)	0.021 (4)	−0.012 (3)	−0.002 (3)	−0.002 (3)
O1	0.025 (3)	0.042 (3)	0.042 (4)	−0.007 (2)	0.002 (2)	0.011 (3)
O2	0.042 (3)	0.028 (3)	0.036 (4)	0.001 (3)	0.013 (3)	0.004 (3)
O3	0.033 (3)	0.035 (3)	0.032 (3)	−0.012 (3)	−0.017 (3)	0.008 (3)
O4	0.038 (3)	0.037 (3)	0.050 (4)	−0.017 (3)	−0.004 (3)	−0.003 (3)
W1	0.03339 (12)	0.03505 (16)	0.03134 (19)	0.0016 (3)	−0.00019 (12)	−0.0005 (3)
W2	0.03320 (13)	0.03300 (16)	0.03245 (19)	−0.0027 (3)	0.00128 (11)	−0.0007 (2)

### Geometric parameters (Å, °)

**Table:**

C1—N1	1.143 (10)	C33—H33	0.9300
C1—W1	2.173 (8)	C34—N23	1.373 (13)
C2—N2	1.154 (10)	C34—C35	1.400 (12)
C2—W1	2.167 (8)	C35—C36	1.308 (13)
C3—N3	1.159 (10)	C35—H35	0.9300
C3—W1	2.145 (7)	C36—N24	1.382 (11)
C4—N4	1.175 (10)	C36—H36	0.9300
C4—W1	2.140 (8)	C37—N25	1.379 (11)
C5—N5	1.183 (10)	C37—C38	1.380 (12)
C5—W1	2.146 (9)	C37—H37	0.9300
C6—N6	1.162 (10)	C38—C39	1.381 (12)
C6—W1	2.159 (8)	C38—H38	0.9300
C7—N7	1.162 (10)	C39—N26	1.355 (11)
C7—W1	2.133 (8)	C39—C40	1.399 (12)
C8—N8	1.134 (11)	C40—C41	1.354 (11)
C8—W1	2.159 (9)	C40—H40	0.9300
C9—N9	1.174 (10)	C41—N25	1.390 (10)
C9—W2	2.119 (7)	C41—H41	0.9300
C10—N10	1.132 (10)	C42—C43	1.289 (12)
C10—W2	2.168 (8)	C42—N28	1.344 (10)
C11—N11	1.076 (11)	C42—H42	0.9300
C11—W2	2.225 (9)	C43—C44	1.483 (13)
C12—N12	1.115 (10)	C43—H43	0.9300
C12—W2	2.200 (8)	C44—N27	1.285 (12)
C13—N13	1.131 (10)	C44—C45	1.349 (11)
C13—W2	2.183 (8)	C45—C46	1.379 (11)
C14—N14	1.141 (10)	C45—H45	0.9300
C14—W2	2.202 (8)	C46—N28	1.449 (11)
C15—N15	1.125 (10)	C46—H46	0.9300
C15—W2	2.172 (8)	C47—N29	1.384 (12)
C16—N16	1.134 (11)	C47—C48	1.404 (13)
C16—W2	2.152 (9)	C47—H47	0.9300
C17—C18	1.360 (11)	C48—C49	1.400 (11)
C17—N17	1.402 (10)	C48—H48	0.9300
C17—H17	0.9300	C49—N30	1.300 (12)
C18—C19	1.421 (11)	C49—C50	1.375 (12)
C18—H18	0.9300	C50—C51	1.343 (12)
C19—N18	1.349 (12)	C50—H50	0.9300
C19—C20	1.385 (11)	C51—N29	1.387 (10)
C20—C21	1.387 (11)	C51—H51	0.9300
C20—H20	0.9300	C52—C53	1.379 (12)
C21—N17	1.382 (11)	C52—N32	1.417 (11)
C21—H21	0.9300	C52—H52	0.9300
C22—N20	1.355 (12)	C53—C54	1.397 (11)
C22—C23	1.361 (12)	C53—H53	0.9300
C22—H22	0.9300	C54—N31	1.316 (12)
C23—C24	1.381 (11)	C54—C55	1.503 (13)
C23—H23	0.9300	C55—C56	1.282 (12)
C24—N19	1.394 (13)	C55—H55	0.9300
C24—C25	1.426 (13)	C56—N32	1.339 (10)
C25—C26	1.273 (12)	C56—H56	0.9300
C25—H25	0.9300	N18—N19	1.278 (9)
C26—N20	1.382 (10)	N20—H20A	0.8600
C26—H26	0.9300	N21—H21A	0.8600
C27—C28	1.290 (11)	N22—N23	1.303 (9)
C27—N21	1.409 (10)	N24—H24	0.8600
C27—H27	0.9300	N25—H25A	0.8600
C28—C29	1.454 (12)	N26—N27	1.257 (8)
C28—H28	0.9300	N28—H28A	0.8600
C29—C30	1.369 (11)	N30—N31	1.303 (10)
C29—N22	1.383 (12)	N32—H32A	0.8600
C30—C31	1.450 (12)	O1—H1B	0.8499
C30—H30	0.9300	O1—H1C	0.8501
C31—N21	1.416 (11)	O2—H2B	0.8456
C31—H31	0.9300	O2—H2C	0.8576
C32—N24	1.367 (12)	O3—H3B	0.8499
C32—C33	1.396 (12)	O3—H3C	0.8499
C32—H32	0.9300	O4—H4B	0.8501
C33—C34	1.382 (11)	O4—H4C	0.8500
			
N1—C1—W1	177.8 (7)	N30—C49—C50	124.5 (8)
N2—C2—W1	175.2 (7)	N30—C49—C48	114.9 (8)
N3—C3—W1	176.5 (8)	C50—C49—C48	120.5 (9)
N4—C4—W1	179.0 (7)	C51—C50—C49	122.0 (8)
N5—C5—W1	176.1 (7)	C51—C50—H50	119.0
N6—C6—W1	175.5 (7)	C49—C50—H50	119.0
N7—C7—W1	175.5 (8)	C50—C51—N29	117.8 (8)
N8—C8—W1	177.3 (7)	C50—C51—H51	121.1
N9—C9—W2	178.0 (8)	N29—C51—H51	121.1
N10—C10—W2	177.6 (8)	C53—C52—N32	117.1 (8)
N11—C11—W2	179.0 (7)	C53—C52—H52	121.5
N12—C12—W2	177.9 (7)	N32—C52—H52	121.5
N13—C13—W2	177.6 (8)	C52—C53—C54	122.9 (9)
N14—C14—W2	178.2 (7)	C52—C53—H53	118.5
N15—C15—W2	178.3 (8)	C54—C53—H53	118.5
N16—C16—W2	172.3 (7)	N31—C54—C53	120.0 (8)
C18—C17—N17	119.7 (8)	N31—C54—C55	125.0 (8)
C18—C17—H17	120.2	C53—C54—C55	115.0 (8)
N17—C17—H17	120.2	C56—C55—C54	120.0 (8)
C17—C18—C19	120.0 (8)	C56—C55—H55	120.0
C17—C18—H18	120.0	C54—C55—H55	120.0
C19—C18—H18	120.0	C55—C56—N32	123.8 (9)
N18—C19—C20	116.4 (8)	C55—C56—H56	118.1
N18—C19—C18	122.9 (8)	N32—C56—H56	118.1
C20—C19—C18	120.4 (8)	C21—N17—C17	120.2 (7)
C19—C20—C21	118.8 (8)	N19—N18—C19	125.0 (11)
C19—C20—H20	120.6	N18—N19—C24	124.1 (12)
C21—C20—H20	120.6	C22—N20—C26	121.0 (8)
N17—C21—C20	121.0 (7)	C22—N20—H20A	119.5
N17—C21—H21	119.5	C26—N20—H20A	119.5
C20—C21—H21	119.5	C27—N21—C31	117.7 (7)
N20—C22—C23	120.2 (8)	C27—N21—H21A	121.1
N20—C22—H22	119.9	C31—N21—H21A	121.1
C23—C22—H22	119.9	N23—N22—C29	116.2 (11)
C22—C23—C24	118.8 (9)	N22—N23—C34	118.8 (11)
C22—C23—H23	120.6	C32—N24—C36	118.6 (9)
C24—C23—H23	120.6	C32—N24—H24	120.7
C23—C24—N19	117.0 (9)	C36—N24—H24	120.7
C23—C24—C25	118.5 (9)	C37—N25—C41	119.7 (8)
N19—C24—C25	124.4 (8)	C37—N25—H25A	120.1
C26—C25—C24	121.4 (9)	C41—N25—H25A	120.1
C26—C25—H25	119.3	N27—N26—C39	126.3 (10)
C24—C25—H25	119.3	N26—N27—C44	129.6 (11)
C25—C26—N20	120.0 (10)	C42—N28—C46	119.1 (8)
C25—C26—H26	120.0	C42—N28—H28A	120.4
N20—C26—H26	120.0	C46—N28—H28A	120.4
C28—C27—N21	122.4 (9)	C47—N29—C51	123.4 (8)
C28—C27—H27	118.8	C49—N30—N31	123.2 (9)
N21—C27—H27	118.8	N30—N31—C54	125.5 (9)
C27—C28—C29	121.7 (9)	C56—N32—C52	121.1 (8)
C27—C28—H28	119.1	C56—N32—H32A	119.4
C29—C28—H28	119.1	C52—N32—H32A	119.4
C30—C29—N22	109.9 (8)	H1B—O1—H1C	109.5
C30—C29—C28	119.6 (8)	H2B—O2—H2C	109.2
N22—C29—C28	130.1 (8)	H3B—O3—H3C	109.5
C29—C30—C31	117.8 (8)	H4B—O4—H4C	109.5
C29—C30—H30	121.1	C7—W1—C4	142.5 (3)
C31—C30—H30	121.1	C7—W1—C3	111.8 (3)
N21—C31—C30	120.6 (8)	C4—W1—C3	75.8 (3)
N21—C31—H31	119.7	C7—W1—C5	72.9 (3)
C30—C31—H31	119.7	C4—W1—C5	73.9 (3)
N24—C32—C33	120.3 (8)	C3—W1—C5	75.6 (3)
N24—C32—H32	119.8	C7—W1—C6	82.7 (3)
C33—C32—H32	119.8	C4—W1—C6	74.9 (3)
C34—C33—C32	119.1 (9)	C3—W1—C6	146.0 (3)
C34—C33—H33	120.4	C5—W1—C6	80.2 (3)
C32—C33—H33	120.4	C7—W1—C8	76.5 (3)
N23—C34—C33	115.4 (9)	C4—W1—C8	122.6 (3)
N23—C34—C35	125.5 (8)	C3—W1—C8	139.7 (3)
C33—C34—C35	119.0 (8)	C5—W1—C8	140.8 (3)
C36—C35—C34	120.4 (8)	C6—W1—C8	72.2 (3)
C36—C35—H35	119.8	C7—W1—C2	142.7 (3)
C34—C35—H35	119.8	C4—W1—C2	73.8 (3)
C35—C36—N24	122.4 (10)	C3—W1—C2	80.1 (3)
C35—C36—H36	118.8	C5—W1—C2	143.4 (3)
N24—C36—H36	118.8	C6—W1—C2	107.3 (3)
N25—C37—C38	121.8 (8)	C8—W1—C2	73.0 (3)
N25—C37—H37	119.1	C7—W1—C1	74.4 (3)
C38—C37—H37	119.1	C4—W1—C1	139.2 (3)
C37—C38—C39	118.8 (9)	C3—W1—C1	72.0 (3)
C37—C38—H38	120.6	C5—W1—C1	120.0 (3)
C39—C38—H38	120.6	C6—W1—C1	141.8 (3)
N26—C39—C38	115.5 (8)	C8—W1—C1	72.9 (3)
N26—C39—C40	126.0 (8)	C2—W1—C1	76.6 (3)
C38—C39—C40	118.4 (8)	C9—W2—C16	75.7 (3)
C41—C40—C39	123.2 (9)	C9—W2—C10	80.4 (3)
C41—C40—H40	118.4	C16—W2—C10	75.5 (3)
C39—C40—H40	118.4	C9—W2—C15	77.2 (3)
C40—C41—N25	118.1 (9)	C16—W2—C15	74.0 (3)
C40—C41—H41	121.0	C10—W2—C15	145.7 (3)
N25—C41—H41	121.0	C9—W2—C13	114.5 (3)
C43—C42—N28	124.1 (9)	C16—W2—C13	138.7 (3)
C43—C42—H42	117.9	C10—W2—C13	143.7 (3)
N28—C42—H42	117.9	C15—W2—C13	70.2 (3)
C42—C43—C44	118.5 (8)	C9—W2—C12	138.2 (3)
C42—C43—H43	120.8	C16—W2—C12	124.4 (3)
C44—C43—H43	120.8	C10—W2—C12	72.0 (3)
N27—C44—C45	119.0 (9)	C15—W2—C12	139.9 (3)
N27—C44—C43	121.6 (8)	C13—W2—C12	76.0 (3)
C45—C44—C43	119.3 (8)	C9—W2—C14	145.0 (3)
C44—C45—C46	120.6 (9)	C16—W2—C14	73.0 (3)
C44—C45—H45	119.7	C10—W2—C14	106.2 (3)
C46—C45—H45	119.7	C15—W2—C14	79.4 (3)
C45—C46—N28	118.3 (8)	C13—W2—C14	80.8 (3)
C45—C46—H46	120.8	C12—W2—C14	74.4 (3)
N28—C46—H46	120.8	C9—W2—C11	71.6 (3)
N29—C47—C48	117.5 (9)	C16—W2—C11	142.1 (3)
N29—C47—H47	121.2	C10—W2—C11	80.6 (3)
C48—C47—H47	121.2	C15—W2—C11	115.7 (3)
C49—C48—C47	118.8 (9)	C13—W2—C11	74.1 (3)
C49—C48—H48	120.6	C12—W2—C11	73.4 (3)
C47—C48—H48	120.6	C14—W2—C11	142.9 (3)

### Hydrogen-bond geometry (Å, °)

**Table:**

*D*—H···*A*	*D*—H	H···*A*	*D*···*A*	*D*—H···*A*
N20—H20A···O2	0.86	1.83	2.671 (10)	166
N21—H21A···N17^i^	0.86	1.76	2.586 (10)	162
N24—H24···O3	0.86	1.73	2.560 (10)	162
N28—H28A···O4^ii^	0.86	1.84	2.670 (10)	161
N32—H32A···O1^iii^	0.86	1.88	2.719 (10)	166
O2—H2B···N3^iv^	0.85	2.47	2.914 (10)	113
O1—H1B···N7	0.85	2.58	3.076 (10)	119
O4—H4C···N4^v^	0.85	2.25	2.845 (9)	127
O3—H3C···N14^v^	0.85	2.50	2.960 (9)	115
O4—H4B···N10^vi^	0.85	2.47	2.913 (9)	114

Symmetry codes: (i) *x*−1/2, −*y*+3/2, −*z*; (ii) *x*, *y*+1, *z*; (iii) *x*, *y*+1, *z*+1; (iv) −*x*+1, *y*−1/2, −*z*+1/2; (v) −*x*+3/2, −*y*+1, *z*+1/2; (vi) *x*−1/2, −*y*+3/2, −*z*+1.

## References

[bb1] Brandenburg, K. (2006). *DIAMOND* Crystal Impact GbR, Bonn, Germany.

[bb2] Bruker (2004). *SMART*, *SAINT* and *SADABS* Bruker AXS Inc., Madison, Wisconsin, USA.

[bb3] Chelebaeva, E., Larionova, J., Guari, Y., Ferreira, R. A. S., Carlos, L. D., Paz, F. A. A., Trifonov, A. & Gueŕin, C. (2008). *Inorg. Chem* **47**, 775–777. 10.1021/ic702192k 18186631

[bb4] Flack, H. D. (1983). *Acta Cryst.* A**39**, 876–881.

[bb5] Ikeda, S., Hozumi, T., Hashimoto, K. & Ohkoshi, S. I. (2005). *Dalton Trans* pp. 2120–2123. 10.1039/b503426d 15957053

[bb6] Kosaka, W., Hashimoto, K. & Ohkoshi, S. I. (2007). *Bull. Chem. Soc. Jpn*, **80**, 2350–2356.

[bb7] Liu, W.-Y., Zhou, H. & Yuan, A.-H. (2008). *Acta Cryst.* E**64**, m1151. 10.1107/S160053680802494X PMC2960576 21201604

[bb8] Matoga, D., Mikuriya, M., Handa, M. & Szklarzewicz, J. (2005). *Chem. Lett* **34**, 1550–1551.

[bb9] Przychodzeń, P., Pełka, R., Lewiński, K., Supel, J., Rams, M., Tomala, K. & Sieklucka, B. (2007). *Inorg. Chem* **46**, 8924–8938. 10.1021/ic700795q 17845030

[bb10] Sheldrick, G. M. (2008). *Acta Cryst.* A**64**, 112–122. 10.1107/S0108767307043930 18156677

[bb11] Wang, Z. X., Shen, X. F., Wang, J., Zhang, P., Li, Y. Z., Nfor, E. N., Song, Y., Ohkoshi, S. I., Hashimoto, K. & You, X. Z. (2006). *Angew. Chem. Int. Ed* **45**, 3287–3291. 10.1002/anie.200600455 16607666

